# Implementation strategies, facilitators, and barriers to scaling up and sustaining demand generation in family planning, a mixed-methods systematic review

**DOI:** 10.1186/s12905-023-02735-z

**Published:** 2023-11-07

**Authors:** Ashraf Nabhan, Rita Kabra, Alyaa Ashraf, Fatma Elghamry, James Kiarie, Family Planning Research Collaborators, Emry Atwa, Samhaa Bahnasy, Marwa Elgendi, Amal Elshabrawy, Salma Eltayeb, Sara Galal, Mariam Kodsy, Nada Makram, Nourhan Mostafa, Noha Sakna, Mohamed Salama, Marwa Snosi, Nouran Wagih, Ahmed Zenhom

**Affiliations:** 1 Department of Obstetrics and Gynecology, Faculty of Medicine, Ain Shams University, Ramses Street, Cairo, Egypt; 2 Department of Sexual and Reproductive Health including UNDP/UNFPA/ UNICEF/WHO/World Bank Special Programme of Research, Development and Research Training in Human Reproduction, World Health Organization, Geneva, Switzerland; 3 Faculty of Medicine, Ain Shams University, Cairo, Egypt

**Keywords:** Family planning, Demand generation, Scaling-up

## Abstract

**Background:**

Demand generation aims to increase clients’ desire to use family planning. The aim of this work was to systematically summarize strategies, facilitators, and barriers to scaling up and sustaining demand generation in family planning.

**Methods:**

We searched electronic bibliographic databases from inception to October 2022. We included quantitative, qualitative, and mixed methods reports on demand generation strategies in family planning, regardless of country, language, publication status, or methodological limitations. We assessed abstracts, titles and full-text papers according to the inclusion criteria, extracted data, and assessed methodological quality of included reports. We used the convergent integrated approach and a deductive thematic synthesis to summarize demand generation themes and subthemes. We used the health system building blocks to synthesize the factors affecting implementation (barriers and facilitators). We used GRADE-CERQual to assess our confidence in the findings.

**Results:**

Forty-six studies (published 1990–2022) were included: forty-one quantitative, one qualitative, and four mixed methods). Three were from one high-income country, and forty three from LMIC settings. Half of reports were judged to be of unclear risk of bias. There were unique yet interrelated strategies of scaling-up demand generation for family planning. Interpersonal communication strategies increase adoption and coverage of modern contraceptive methods, but the effect on sustainability is uncertain. Mass media exposure increases knowledge and positive attitudes and may increase the intention to use modern contraceptive methods. Demand-side financing approaches probably increase awareness of contraceptives and the use of modern contraceptive methods among poor clients. Multifaceted Demand generation approaches probably improve adoption, coverage and sustainability of modern methods use. Factors that influence the success of implementing these strategies include users knowledge about family planning methods, the availability of modern methods, and the accessibility to services.

**Conclusions:**

Demand generation strategies may function independently or supplement each other. The myriad of techniques of the different demand generation strategies, the complexities of family planning services, and human interactions defy simplistic conclusions on how a specific strategy or a bundle of strategies may succeed in increasing and sustaining family planning utilization.

**Trial Registration:**

Systematic review registration: Center for Open Science, osf.io/286j5

## Abstract (plain language summary)

Family planning could prevent one third of maternal deaths by allowing women to delay motherhood, avoid unintended pregnancies and subsequent abortions. Demand generation is one of the critical factors for increasing coverage and sustainability of family planning programs. Demand generation activities aim to increase clients’ desire to use family planning by changing their attitudes or perceptions about FP or increasing their awareness or knowledge about FP methods and also by improving access to contraceptive services. Many demand generation activities also aim to shift social and cultural norms to affect individual behavior change. Scaling up demand generation and ensuring that demand for family planning is satisfied is essential for achieving universal access to reproductive health-care services. We systematically searched for and summarized reports of strategies to scale up demand generation for family planning. Available evidence shows that interpersonal communication strategies increase adoption and coverage of modern contraceptive methods, but the effect on sustainability is uncertain. Mass media exposure increases knowledge and positive attitudes and may increase the intention to use modern contraceptive methods. The effect of new media is uncertain. Demand-side financing approach probably increase awareness of contraceptives and the use of modern contraceptive methods among poor women. The most apparent factors influencing the success of implementing these strategies include knowledge about family planning methods, especially regarding side effects and health concerns, the availability of modern contraceptive methods, and the accessibility to family planning services.

## Background

Improving the effectiveness of family planning (FP) programs is critical for empowering women and adolescent girls, improving human capital, reducing dependency ratios, reducing maternal and child mortality, and achieving demographic dividends particularly in low- and middle-income countries. Family planning could prevent one third of maternal deaths by allowing women to delay motherhood, avoid unintended pregnancies and subsequent abortions [1, 2].

One of the driving forces for increasing coverage and sustainability of family planning programs is demand generation. Demand generation strategies encompass three categories: interpersonal communications, mass media, and innovative financing approaches. Demand generation activities aim to increase clients’ desire to use family planning by changing their attitudes or perceptions about FP or increasing their awareness or knowledge about FP methods and also by improving access to contraceptive services. Many demand generation activities also aim to shift social and cultural norms to affect individual behavior change [3, 4].

Implementing demand generation strategies and ensuring that demand for family planning is satisfied are essential for achieving universal access to reproductive health-care services, as called for in the 2030 Agenda for Sustainable Development [5]. Scaling up is defined as deliberate efforts to increase the impact of health service innovations successfully tested in pilot or experimental projects so as to benefit more people and to foster policy and program development on a lasting basis [6–9].

Therefore, as part of its family planning strategy, the WHO has commissioned this systematic review of scaling up demand generation in family planning.

The overall aim of the review was to describe and assess the quality of the evidence on scaling up demand generation in family planning. The review has the following objectives:

- to identify, appraise and synthesize research evidence regarding the approaches or strategies to scale up demand generation in FP for improving adoption, coverage, and sustainability;
- to identify, appraise and synthesize research evidence on the barriers to and facilitators of scaling up demand generation for family planning.

## Methods

We conducted a systematic review, following the JBI methodology for mixed methods systematic reviews (MMSR) [10] and methods suggested by the Cochrane Effective Practice and Organisation of Care (EPOC) Review Group [11].

The protocol, available as a preprint [12], was registered in the Center for Open Science platform (10.17605/OSF.IO/286J5).

We reported the review according to the Preferred Reporting Items for Systematic Reviews and Meta-Analyses (PRISMA) [13].

### Criteria for considering studies for this review

#### Types of participants

We included all types of participants who are the target of scaling up demand generation for FP.

#### Phenomena of interest

We included studies where the focus is scaling up demand generation for FP.

#### Types of interventions

For this review, we considered demand generation strategies under the following categories: interpersonal communications, mass media, and innovative financing approaches [3].

#### Outcomes

We considered implementation research outcomes mainly adoption (the intention, initial decision, or action to try to employ an intervention; also known as Uptake, Utilization, Intention to use), coverage (the degree to which the population that is eligible to benefit from an intervention actually receives it), and sustainability (the extent to which an intervention is maintained; also known as maintenance, Continuation) [14, 15].

#### Barriers and facilitators (factors that influence demand generation)

The approach to the factors affecting demand generation to scale up FP was based on the SURE (Supporting the Use of Research Evidence) framework [16]. We considered the factors affecting implementation at all levels namely recipients of care, providers of care, other stakeholders (including other healthcare providers, community health committees, community leaders, program managers, donors, policymakers and opinion leaders), health system constraints, and social and political constraints.

The factors were finally grouped by the categories of health system building blocks (HSBB). HSBB is an analytical framework used by WHO to describe health systems, disaggregating them into 6 core components, with people in the center, (i) service delivery, (ii) health workforce, (iii) health information systems, (iv) Medical products, vaccines and technologies (access to essential medicines), (v) financing, and (vi) leadership and governance [17].

#### Types of studies

We included primary quantitative studies, qualitative studies, process evaluation studies, policy analysis studies, and case studies. Mixed method studies were only considered if data from the quantitative or qualitative components can be clearly extracted.

We excluded editorials, commentaries, proposals, conference abstracts and systematic reviews. We also excluded reports that lacked a clear methodology section.

There was no restriction on length of study follow-up, language of publication, or country of origin.

### Literature search

#### Sources

The search strategy aimed to locate both published and unpublished studies. We searched bibliographic databases for peer reviewed publications as well as grey literature. We searched the following electronic bibliographic databases (from inception to 15th September 2022): MEDLINE, PubMed, Scopus, the Cochrane Library, and Global Index Medicus, World Health Organization (www.globalindexmedicus.net).

We also searched gray literature using the search engines and websites of relevant organizations. We hand searched citations in included articles.

#### Search strategy

The search terms were developed in consultation with the other authors using a combination of keywords and Medical Subject Headings (MeSH). The search strategy will be first developed in Pubmed format and was adapted to the other databases. The search strategies for various platforms are available in an open access repository [18].

We will use the following terms ((Implementation Science[MeSH Terms]) OR (“Health Services Needs and Demand”[MeSH Terms]) OR (“demand generation” [Text Word]) OR (“demand side” [Text Word])) AND ((Family Planning Services[MeSH Terms]) OR (contraception[MeSH Terms]) OR (contracept*[Text Word]) OR (“family planning”[Text Word]) OR (“birth control”[Text Word]) OR (“birth spacing”[Text Word])). We aimed at sensitivity rather than precision since we opted to minimize false negative results.

#### Management of search results

All search results were imported into Jabref v5. Duplicate search results were identified by the software and were eliminated after being revised by the authors, using a method that enables retaining unique citations without accidentally excluding false duplicates.

### Data collection

#### Study selection

We developed a study selection form based on our eligibility criteria. After removal of duplicates, two review authors independently piloted the study selection form with a small random sample of studies to assess understanding of eligibility criteria and ease of use of the form. Two review authors independently screened all titles/abstracts and full text to identify the relevant studies. Discrepancies between review authors regarding study eligibility were resolved by consensus or, when required, with a third party. We used the PRISMA flowchart to describe the process of study selection.

### Data extraction

Two review authors independently extracted characteristics from the included studies: study title, first author, year of publication, country of study, the country’s economic status (low-, middle-, or high-income), study type and design. We extracted the demand generation strategies mentioned in each study, the target of the demand generation activity, implementation outcome evaluated in each study, and barriers and facilitators. We resolved any disagreement in the data collection process through discussion and consensus between the two reviewers and, if needed, with a third party.

### Quality assessment

For each included study, the methodological quality were described using the corresponding Mixed-Methods Appraisal Tool (MMAT) criteria [19, 20]. Two independent reviewers assessed the quality of included studies using MMAT, with a third independent reviewer to be used in case of any discrepancies. We accepted that there is no ‘gold standard’ approach for assessing the methodological quality of primary qualitative studies, but believe that MMAT fits the context of this synthesis [19, 20].

We did not exclude studies based on methodological limitations, but rather will use the findings to assess the confidence in the findings.

### Data synthesis

We used the convergent integrated approach. The quantitative data was then converted into “qualitized data.” This involved transformation into textual descriptions or narrative interpretation of the quantitative results in a way that answers the review questions [10].

We grouped articles according to categories of demand generation, as defined above. We used a deductive thematic synthesis using the health system building blocks to synthesize the factors affecting implementation (barriers and facilitators).

### Appraisal of confidence in the review findings

We used GRADE-CERQual to assess the confidence that can be placed in each review finding [21] based on four components: methodological limitations of included studies, coherence of the review findings, adequacy of the data contributing to a review finding, and relevance of the included studies to the review question. After assessing each of the four components, we made a judgement about the overall confidence in the evidence supporting each review finding. All findings start as high confidence and were then graded down if there are important concerns regarding any of the four components. We judged confidence as high, moderate, low, or very low. The final assessment was based on consensus among the review authors. We presented summaries of the findings and our assessments of confidence in these findings in the Summary of findings Table [21].

### Researchers’ reflexivity

We maintained a reflexive stance throughout the stages of the review process, from study selection to data synthesis. The team discussed the Progress regularly and explored critically every step of the work. As a review team, we all have clinical backgrounds. In addition, three review authors have received advanced training in implementation science (AN, RK, JK) and are well versed in relevant theory. Based on our collective and individual experiences (as clinicians, academics and researchers), we anticipated the findings of our review to reveal a combination of organisational, professional and individual factors influencing the demand generation for family planning. We, as a team, remained mindful of our presuppositions and support each other to minimize the risk of these skewing the synthesis or the interpretation of the findings. We kept a reflexive journal throughout the review process in which to document and reflect on progress and decisions made [11].

## Results

### Study selection

**Fig. 1:**
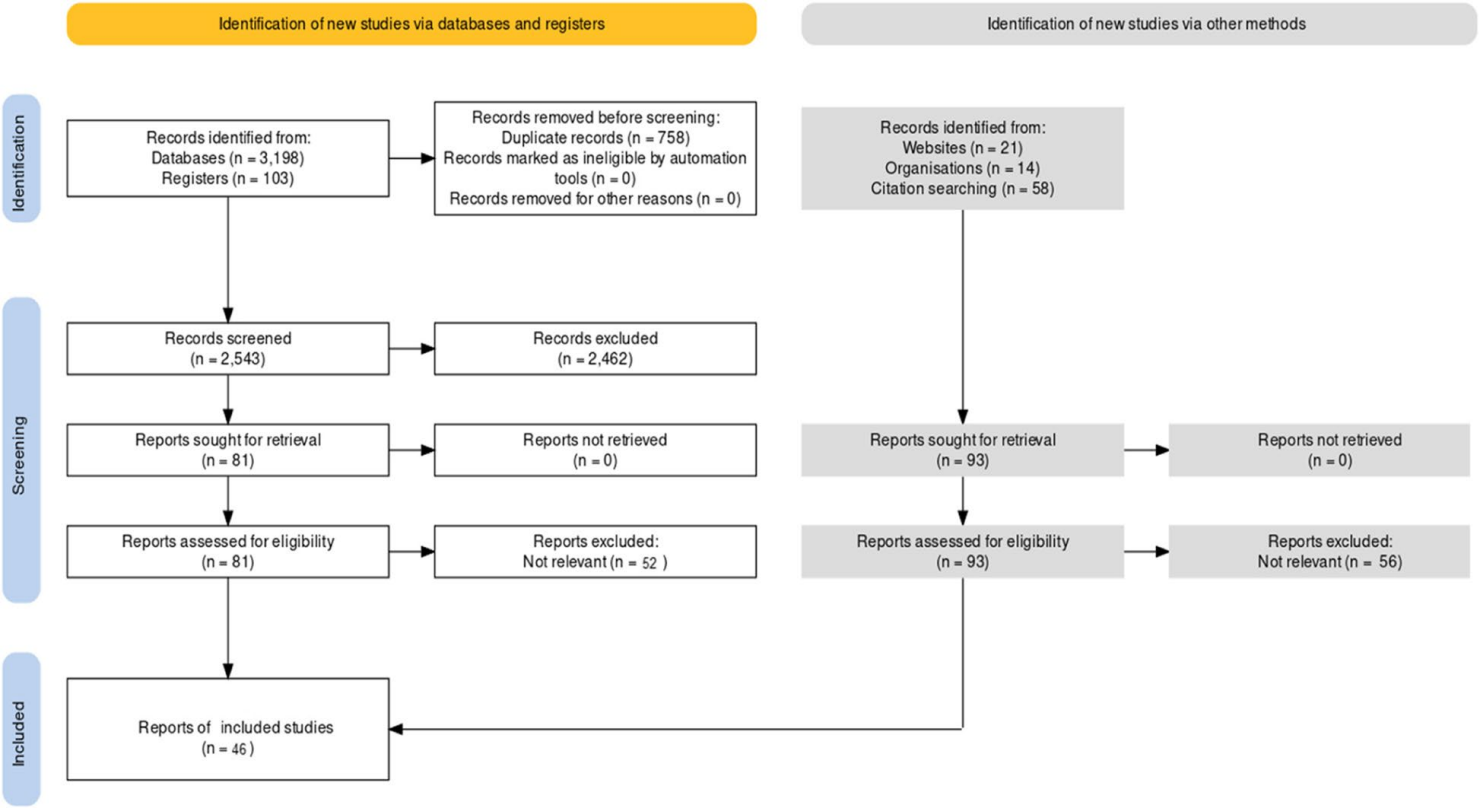
PRISMA Flowchart

The flow of identification, screening, and including 46 reports is depicted in Fig. 1.

### Characteristics of included studies

The 46 included studies used Quantitative (41/46, 89.13%), Qualitative (1/46; 2.17%), and Mixed methods (4/46; 8.70%). The studies were reported from 23 countries from all regions and from Low (Ethiopia, Gambia, Madagascar, Malawi, Mali, Rwanda, Uganda, Yemen,Zambia), Lower middle (Cambodia, Cameroon, Egypt, Ghana, India, Iran, Kenya, Nepal, Nigeria, Pakistan, Senegal, Tanzania), Upper middle (Turkey) and High income (USA) countries, Table 1.

**Table 1:** Number of included reports by country

Country	Number of reports	Income
India	6	Lower middle
Pakistan	6	Lower middle
Kenya	3	Lower middle
Nigeria	3	Lower middle
USA	3	High
Ethiopia	2	Low
Malawi	2	Low
Ghana	2	Lower middle
Nepal	2	Lower middle
Tanzania	2	Lower middle
Gambia	1	Low
Madagascar	1	Low
Mali	1	Low
Rwanda	1	Low
Uganda	1	Low
Yemen	1	Low
Zambia	1	Low
Cambodia	1	Lower middle
Cameroon	1	Lower middle
Egypt	1	Lower middle
Iran	1	Lower middle
Senegal	1	Lower middle
Turkey	1	Upper Middle

### Methodological quality

For each included study, the methodological quality was described using the corresponding MMAT criteria. We judged 14 studies (30.43%) to be of low risk of bias, 10 studies (21.74%) to have a high risk of bias, and 22 studies (47.83%) to have an unclear risk of bias. We did not exclude studies based on methodological limitations, but rather used the findings to assess the confidence in the findings.

### Findings of the review

We used a convergent integrated approach and a deductive thematic synthesis of the different approaches to scale up demand generation for family planning and the factors influencing implementation.

#### Strategies of demand generation in family planning

The review identified unique yet interrelated strategies of scaling-up demand generation for family planning, Table 2.

Interpersonal communications included 16 reports: [22–37]. These included diverse approaches: home visits, one-on-one discussion, small group discussions, lectures, workshops. Community events included street plays and dramas, caravan road shows, community drama/puppet shows, sports competitions, beauty contests, bicycle races, public entertainment events, population weekends, and religious leaders’ speeches. This category included counselling and referral.

Mass- and mid- media included Television, Radio, Wall paintings, Leaflets, Posters, Booklets, Brochures, Newspaper and magazines. New media included mobile messages and social media namely Facebook (advertisements or page) in 8 reports, [38–45].

Demand-side financing strategies included small cash incentives and vouchers in 7 reports [46–52].

Reports used a multifaceted approach, namely Interpersonal communications plus Mass media in 12 reports [53–64], Interpersonal communications plus Financing in 2 reports: [65, 66], and a bundle of Interpersonal communications plus Mass media plus Financing in one report [67].

**Table 2:** Strategies of demand generation in family planning

Theme	Sub-theme	Studies
Interpersonal communications	One-on-One discussion	[22, 54, 55, 42, 33]
	Community events (such as street plays and dramas, caravan road shows, community drama/puppet shows, sports competitions, beauty contests, bicycle races public entertainment events, population weekends)	[54–56, 59, 60]
	Small Group discussions	[55, 23, 42, 27, 28, 63, 35]
	Home visits	[66, 25, 27, 63, 37, 64]
	Religious leaders’ speeches	[28, 60, 32]
	Counselling and referral	[66, 26, 25, 36, 62, 34, 56, 24]
	Lectures, workshops	[29]
Mass media	Leaflets	[22, 66, 60, 30, 33, 62, 59]
	Posters	[66, 59, 60, 30, 62]
	Television	[56, 57, 59, 60, 44, 45, 63]
	Radio	[38, 39, 54–58, 60, 63]
	Wall paintings	[66, 59, 62]
	Booklets	[59, 62]
	Brochures	[58]
	Newspaper and magazines	[38, 39, 56]
	Facebook (advertisements, page)	[41, 43]
	Mobile messages	[57, 64]
Innovative financing interventions	Vouchers	[65, 66, 46, 47, 67, 49–51]
	Small cash incentives	[52]

#### Effect of demand generation strategies

The effect of demand generation strategies were grouped by either reports of unique strategies, Table 3 or multifaceted strategies, Table 4.

Interpersonal communications strategies contributed to 67.39% (31/46) of the reports either independently (Table 3) or as part of a bundle (Table 4) to generate demand for FP.

**Table 3:** Summary of the reports of unique demand generations strategies

Main Demand Generation strategy	Outcomes	Number of studies	Summarized review finding	GRADE-CERQual Assessment
Interpersonal communication	Adoption, Coverage, Sustainability	16	Interpersonal communications increase the use of modern contraceptive methods. The effect on sustainability is uncertain.	Moderate confidence
Mass media	Adoption, coverage	8	Mass media exposure increases knowledge and positive attitudes. Mass media may increase the intention to use modern contraceptive methods. The effect of new media is uncertain.	Moderate confidence
Demand side financing	Awareness, Adoption, Coverage	7	Demand-side financing approach (using vouchers or small cash incentives) probably increases awareness of contraceptives and the use of modern contraceptive methods among poor women.	Moderate confidence

**Table 4:** Summary of the reports of multifaceted demand generations strategies

Main Demand Generation strategies	Outcomes	Number of studies	Summarized review finding	GRADE-CERQual Assessment
Interpersonal communications plus Mass media	Adoption, Coverage, Sustainability	12	Interpersonal communications plus Mass media increase adoption, use and sustainability of modern contraceptive methods among women and among men as well.	Moderate confidence
Interpersonal communications plus mass media plus financing	Awareness, Coverage, Sustainability	2	Interpersonal communications plus financing plus mass media increase the awareness and use of modern contraceptive methods and may promote sustainability	Low confidence
Interpersonal communications plus financing	Adoption, Coverage	1	Interpersonal communications plus financing may increase the intention to use and the actual use of contraceptive methods, particularly in people below poverty line.	Very Low confidence

### Factors influencing demand generation for family planning

The health system building blocks frame work was used for the synthesis of factors that influence demand generation for family planning.

#### People

- Knowledge about family planning methods, especially regarding side effects and health concerns [32, 42, 56, 57].
- Woman’s preference and acceptability [42, 57].
- Engagement of partners in discussing family planning [25, 57].
- Interest to discuss family planning [39, 42].
- Motivation to use family planning [22].
- Reach of mass media [44, 54].
- Social acceptability to approach unmarried women to discuss contraception [25].
- Traditional and religious beliefs regarding the number of children [24].

#### Financing

- Affordability of family planning services [25].
- Financial benefits associated with practicing family planning [35].

#### Health workforce

- Providers’ age, gender, and religion [25, 35].
- Family planning nurses and community healthcare workers sharing sound knowledge [56].
- Number of Health workforce, especially female healthcare workers [24, 32].

#### Leadership and governance

- Concurrent multiple demand generation programs within the same area in need [25].
- Consistency of implementing family planning programs [25].
- Degree of reliance on donor driven management and funding [25].
- Endorsement of family planning by the government [56].
- Integration of community-based health workers into healthcare system [25].
- Integration of non-governmental organizations trained field workers into the healthcare system [25].

#### Medical products

- Availability of modern contraceptive methods [24, 32, 35, 54].
- Number of methods available to women [54].

#### Service delivery

- Accessibility to family planning services [24, 35, 54].
- Ease of use of the family planning method [55].

## Discussion

### Summary of the evidence

Demand-side unmet need (lack of demand for contraception), compared with supply-side unmet need, is responsible for the vast majority of total unmet need, ranging from 69 to 84% of unmet need [68]. This implies that a significant proportion of nonusers, irrespective of their age or level of education, consciously and knowingly decide against using modern family planning methods, even when they are not actively seeking to conceive [68–70]. Therefore, it was imperative to critically summarize best available evidence to understand what and how demand generation strategies can help for improving family planning services and reducing unmet need.

In the current review, demand generation strategies in cross-cutting themes were identified within the included studies. Available evidence suggests that scaling up demand generation using interpersonal communication strategies can increase adoption and coverage of modern contraceptive methods, but the effect on sustainability is uncertain. Demand generation through mass media exposure increases knowledge and positive attitudes and may increase the intention to use modern contraceptive methods. The effect of new media is uncertain. Demand-side financing approach probably increases awareness and use of modern contraceptive methods among poor women. The results are in agreement with previous reviews that examined demand generation strategies and how these might improve adoption and coverage of family planning methods [71–73].

Our results indicate that demand generation strategies may function independently or supplement each other. Each theme seems to improve certain aspect contributing to scaling up family planning. Evidence for sustainability is insufficient and this remains an important issue for countries striving to maintain a reduction in unmet needs and improvement of contraceptive prevalence rates. The need for integration with health system is critical for family planning to be institutionalized and therefore sustainable [74].

The heterogeneity in the designs of studies assessing demand-side interventions and the lack of evidence on indicators used to measure the outcomes of such interventions make it difficult to draw overall conclusions about the strategies that can be scaled up. These issues have been observed, yet remain an resolved challenge [63].

There is lack of reports on social media (one platform in two reports) and cellular phone technology (two reports) for demand generation in family planning. Social media allows users to connect in a virtual network or community, facilitating reach and usability of shared information. Types of social media include social networks (e.g., Facebook, Twitter), video sharing (e.g., YouTube), photo-sharing platforms (e.g., Instagram), or messaging apps (e.g., WhatsApp, Telegram). In each of these types, the over-arching characteristics include: connections and relationship-building, speedy delivery, and not limited by geography. This issue is extremely important in the currrent era since there is evidence that incorporating social media features into social behavior change activities has been shown to contribute positively to their success [75].

In the current synthesis of barriers to the success of demand generation strategies included knowledge about family planning methods, especially regarding side effects and health concerns. The significance of providing precise information to individuals for making informed health decisions cannot be overstated [76]. The discussion of an individual’s lack of demand for contraceptive methods that health providers consider advantageous always necessitates a consideration of health literacy. The significance of health literacy is important, specifically in relation to the prevalence of “myths and rumors” as justifications for the non-utilization of contraceptives [77, 78]. Women’s concerns regarding the potential adverse effects of contraceptives on their bodies are valid. These valid concerns are conceptualized as a demand-side rationale for abstaining from contraceptive use. Particularly for those women whose inclination to avoid pregnancy is weak, the urge to avert contraceptive side effects may emerge as a more persuasive impetus, thereby culminating in non-utilization of contraceptives [79]. Although the concerns regarding the adverse effects of contraception is frequently associated with myths and misconceptions, an examination of women’s views revealed that individuals who attribute their non-use to health concerns are more likely to have previously used modern contraceptive method [80]. In the current era, it is critical to scrutinize the systematic exclusion of women’s voices from the debate concerning their bodies and families.

### Limitations

Although we took every effort to minimize the potential for biases in the review process, sources of potential bias may exist. First, while our searches were comprehensive, there is a possibility that some relevant studies were missed for assessment by the review since the results of some programs may have not been made public.

Second, a potential bias in reviews in this area is the adoption of clear criteria for what constitutes a standalone demand side strategy, which is never the case.

Third, each theme of demand-generation contains a diversity of possible processes, content, and operational environments. Because these variables are often not controlled across studies, it is difficult to rigorously determine the situations in which specific strategies work best. Despite this, the strengths of different strategies in different circumstances can still be realized.

Finaly, information regarding the processes of demand generation strategies in the included reports were not described in sufficiently informative details.

## Conclusions

Demand generation strategies may function independently or supplement each other. Each category possibly improves certain aspect contributing to improving awareness, adoption, and use of modern contraceptive methods. Evidence for sustainability is insufficient. The myriad of techniques of the different demand generation strategies, the complexities of programs, and human interactions defy simplistic conclusions on how a specific strategy or a bundle of strategies may succeed in scaling up demand generation thus increasing use and sustaining family planning services.

## Data Availability

All data generated or analysed during this study are included in this published article and its supplementary information files.
